# Role of IL13RA2 in Sunitinib Resistance in Clear Cell Renal Cell Carcinoma

**DOI:** 10.1371/journal.pone.0130980

**Published:** 2015-06-26

**Authors:** Noboru Shibasaki, Toshinari Yamasaki, Toru Kanno, Ryuichiro Arakaki, Hiromasa Sakamoto, Noriaki Utsunomiya, Takahiro Inoue, Tatsuaki Tsuruyama, Eijiro Nakamura, Osamu Ogawa, Tomomi Kamba

**Affiliations:** 1 Department of Urology, Graduate School of Medicine, Kyoto University, Kyoto, Japan; 2 Department of Diagnostic Pathology, Graduate School of Medicine, Kyoto University, Kyoto, Japan; 3 Laboratory for Malignancy Control Research, Medical Innovation Center, Graduate School of Medicine, Kyoto University, Kyoto, Japan; Children's Hospital Boston & Harvard Medical School, UNITED STATES

## Abstract

Vascular endothelial growth factor (VEGF) and mammalian target of rapamycin are well-known therapeutic targets for renal cell carcinoma (RCC). Sunitinib is an agent that targets VEGF receptors and is considered to be a standard treatment for metastatic or unresectable clear cell RCC (ccRCC). However, ccRCC eventually develops resistance to sunitinib in most cases, and the mechanisms underlying this resistance are not fully elucidated. In the present study, we established unique primary xenograft models, KURC1 (Kyoto University Renal Cancer 1) and KURC2, from freshly isolated ccRCC specimens. The KURC1 xenograft initially responded to sunitinib treatment, however finally acquired resistance. KURC2 retained sensitivity to sunitinib for over 6 months. Comparing gene expression profiles between the two xenograft models with different sensitivity to sunitinib, we identified interleukin 13 receptor alpha 2 (IL13RA2) as a candidate molecule associated with the acquired sunitinib-resistance in ccRCC. And patients with high IL13RA2 expression in immunohistochemistry in primary ccRCC tumor tends to have sunitinib-resistant metastatic site. Next, we showed that sunitinib-sensitive 786-O cells acquired resistance *in vivo* when IL13RA2 was overexpressed. Conversely, shRNA-mediated knockdown of IL13RA2 successfully overcame the sunitinib-resistance in Caki-1 cells. Histopathological analyses revealed that IL13RA2 repressed sunitinib-induced apoptosis without increasing tumor vasculature *in vivo*. To our knowledge, this is a novel mechanism of developing resistance to sunitinib in a certain population of ccRCC, and these results indicate that IL13RA2 could be one of potential target to overcome sunitinib resistance.

## Introduction

Renal cell carcinoma (RCC) accounts for 2–3% of all malignancies in adults and causes approximately 102,000 deaths worldwide each year [1]. Approximately 30% of newly diagnosed patients present with locally advanced and/or metastatic RCC [2, 3]. Treatment of metastatic RCC is difficult because it shows limited or no responsiveness to conventional anticancer therapies such as radiation, chemotherapy, and cytokine therapy [4].

Recent increased understanding of the underlying molecular biology of RCC has established the vascular endothelial growth factor (VEGF) and mammalian target of rapamycin (mTOR) pathways as relevant therapeutic targets [5]. Agents targeting the VEGF receptor and mTOR complex 1 (mTORC1) are more effective than traditional cytokine therapy and improve the prognosis of RCC patients [6].

Sunitinib is a small molecule inhibitor of multiple receptor tyrosine kinases (RTKs), including VEGF receptors (VEGFR-1, VEGFR-2, and VEGFR-3), platelet-derived growth factor receptors (PDGFR-α and PDGFR-β), fms-related tyrosine kinase 3 (FLT3), stem cell growth factor receptor KIT, and RET [7, 8], and is currently considered as a standard first-line treatment for metastatic clear cell RCC (ccRCC) [9, 10]. Sunitinib as first-line treatment of patients with metastatic RCC resulted in longer overall survival and progression-free survival compared with patients treated with conventional drugs [9–11]. However, despite the efficacy of sunitinib, ccRCC often develops resistance to sunitinib, and the majority of patients who receive sunitinib for treatment of advanced ccRCC exhibit progressive disease after 1 year of treatment [5]. Several hypotheses have been proposed regarding the mechanism underlying resistance to sunitinib [12, 13], but they have not yet been fully elucidated.

Previous reports indicated that primary xenograft models directly transplanted from patient surgical specimens can recapitulate the original clinical course because these implanted tumor grafts contain both epithelial and stromal cells that may co-proliferate to form histologically complex tumors in host mice [14, 15]. Therefore, in this study, we aimed to clarify the mechanisms underlying resistance to sunitinib by establishing such primary xenograft models from patient-derived tumor tissues and to identify potential targets to overcome sunitinib resistance.

## Materials and Methods

### Patients and RCC samples

RCC tumor specimens were obtained at the Department of Urology, Kyoto University Hospital with appropriate written informed consent. This study was approved by the Kyoto University’s institutional review board (IRB approved number G52: Research about an individualized treatment for urological cancer using gene profiling).

### Cell culture

786-O and Caki-1 cell lines were purchased from the American Type Culture Collection (Rockville, MD). Cells were cultured routinely in Dulbecco's Modified Eagle Medium (Invitrogen, Carlsbad, CA) containing 10% fetal bovine serum supplemented with 1% penicillin/streptomycin. Caki-1 cells stably infected with shRNA lentivirus and 786-O cells stably infected with pBABE-puro retroviruses were selected in the presence of 1.5 μg/ml puromycin.

### Generation of xenograft models

All experiments involving laboratory animals were performed in accordance with the Guidelines for Animal Experiments of Kyoto University (Permit Number 13336: Research of the novel mechanisms developing resistance to anti-angiogenic treatment in renal cell carcinoma). To establish primary xenograft models, local tumors of the kidney were resected by radical nephrectomy, minced into 20–30 mm^3^ fragments, and subcutaneously transplanted into 5-week-old CB-17/Icr-crj SCID mice (Charles River, Yokohama, Japan) on the day of surgery. KURC1 (Kyoto University Renal Cancer 1) and KURC2 xenografts were established within 3–4 months after the first inoculation. Xenograft tumors were extracted and transplanted into several SCID mice.

To establish cell line xenograft models, a total of 1.0×10^7^ cells were subcutaneously injected into both flanks of 5–6-week-old female BALB/cA Jcl nude(nu/nu) mice (CLEA, Tokyo, Japan) and tumor volumes were measured twice a week. All experiments were performed under sodium pentobarbital anesthesia, and all efforts were made to minimize suffering. Following the experimental procedures, all animals were euthanized by carbon dioxide and tumors were excised.

### *In vivo* sunitinib administration

Sunitinib provided by Pfizer Global Pharmaceuticals was diluted in dimethyl sulfoxide (DMSO). Sunitinib treatment for primary or cell line xenograft tumors was commenced from the fourth week after transplantation. Forty mg/kg sunitinib was orally administered once a day for the treatment group, and vehicle only was given to the control group. Affected tumors were considered resistant when the tumor growth rate was comparable to that of the control tumor. Tumors were resected 24 hours later from the last sunitinib or vehicle administration. Because of the difference of growth rate and sunitinib response between 786-O and Caki-1 xenograft tumors[16, 17], we needed to observe for a longer time in Caki-1 xenograft experiment than in 786-O, consequently sacrificed at different time points with each cell lines.

### Antibodies and reagents

Antibodies were purchased commercially as follows: human IL13RA2 (MAB614 for immunoblotting and AF146 for immunohistochemistry, R&D Systems, Minneapolis, MN), human STAT6, human phospho-STAT6 Y641 (Cell Signaling Technology, Beverly, MA), human ssDNA (IBL, Tokyo, Japan), human β-actin, human N-cadherin and mouse CD31 (Abcam, Cambridge, MA).

### Immunohistochemistry

Immunohistochemical analysis was performed on formalin-fixed, paraffin-embedded 17 clinical ccRCC samples or xenograft tissues as described previously [18]. The degree of IL13RA2 immunopositivity in clinical samples was assessed as none, weak, or strong, and the assessment was performed with no prior knowledge of the response to sunitinib. Tumor microvessel density (MVD) was evaluated using CD31 as an endothelial marker. MVD was determined in 3 fields of each sample according to the method of Weidner [19]. Apoptosis was evaluated by counting the ssDNA-positive nuclei rate. Numbers of total or ssDNA-positive apoptotic nuclei were automatically quantitated in 3 fields of each sample using ImageJ software (National Institutes of Health, Bethesda, MD). The ssDNA-positive rate was calculated as the percentage of positive tumor nuclei divided by the total number of tumor nuclei examined.

### Protein extraction and immunoblot analysis

Whole cell proteins were isolated from snap-frozen specimens or cultured cells and analyzed by immunoblotting as previously described [20, 21].

### Quantitative real-time PCR (qPCR)

Total RNA was extracted from vehicle-treated control, sunitinib-treated sensitive or resistant tumors of KURC1, KURC2, and cell line subclones, as previously described [22]. cDNA was synthesized from total RNA using a ReverTra Ace qPCR RT Kit (Toyobo, Osaka, Japan). qPCR was performed using SYBR green PCR Master Mix (Applied Biosystems, Foster City, CA) and monitored using GeneAmp 5700 (Applied Biosystems). PCR reactions were performed in triplicate. The thermal cycling conditions were 95°C for 15 s, 60°C for 30 s, and 72°C for 30 s. The values were normalized to the levels of amplified glyceraldehyde-3-phosphate dehydrogenase (*GAPDH*). The primer sequences were as follows: *GAPDH*, 5′-GAAGGTGAAGGTCGGAGTC-3′ (sense) and 5′-GAAGATGGTGATGGGATTTC-3′ (antisense); and *IL13RA2*, 5′-TTGCTTGGCTATCGGATGCT-3′ (sense) and 5′-GGGTTAACTTTTATCTCGGTGTCTGA-3′ (antisense).

### Microarray analysis

For microarray experiments, we used total RNA extracted from two resistant, one sensitive, and two control KURC1 tumors, and one sensitive and one control KURC2 tumor. Microarray analyses were performed at Genetic Lab Co., Ltd. (Sapporo, Japan) using GeneChip Human Gene 1.0 ST Arrays (Affymetrix, Santa Clara, CA). The results were analyzed using GeneSpring GX (Agilent, Santa Clara, CA) by Genetic Lab Co., Ltd. Our microarray data was registered at the NCBI GEO (GSE66346).

### Lentiviruses

Lentivirus-based plasmids containing pLKO.1 shRNA sets to human IL13RA2 (RHS4533-NM000640; TRCN0000058523-27) were purchased from Open Biosystems (Huntsville, AL). Non-silencing shRNAs (Open Biosystems) were used as negative controls. The experimental procedure for shRNA transfection was performed according to the Open Biosystems technical manual.

### Plasmid construction and retroviral expression

Full-length IL13RA2 cDNA was amplified by PCR from Caki-1 cell cDNA using PrimeSTAR HS DNA polymerase (Takara Bio, Shiga, Japan) and cloned into the pBABE-puro retroviral vector. The oligonucleotide sequences used in the construction of the expression construct were as follows: *IL13RA2*, 5′-CACCATGTATCCATATGATGTTCCAGATTATGCTGGATCCGCTTTCGTTTGCTTGGCTATCGGATGCTTATATAC-3′ and 5′-ACACCCTAACTGACACACATTCCCAGGGTCGACTCATGTATCACAGAAAAATTCTGGAATCATTTTTG-3′. The PCR products were inserted into pBABE-puro vector at BamH1/Xho1 sites using an Infusion-HD Cloning Kit (Takara Bio, Shiga, Japan). G3T-hi packaging cells were infected with retroviral plasmids using a Retrovirus Packaging Kit (Ampho, Takara Bio). These experiments were performed according to the manufacturer’s instructions.

### Oncomine database analysis

Oncomine data was obtained from Vasselli et al. [23] and Bittner. Expression levels of IL13RA2 were compared with tumor grade and prognosis in ccRCC.

### Statistical analysis

Data are expressed as the mean ± SE. The significance of differences between means was assessed using the Student’s *t*-test. *In vitro* cell proliferation and tumor growth *in vivo* were analyzed by two-way repeated ANOVA. *P* values of < 0.05 were considered statistically significant.

## Results

### Establishment of the primary RCC xenograft model and acquisition of resistance to sunitinib

**Fig 1:**
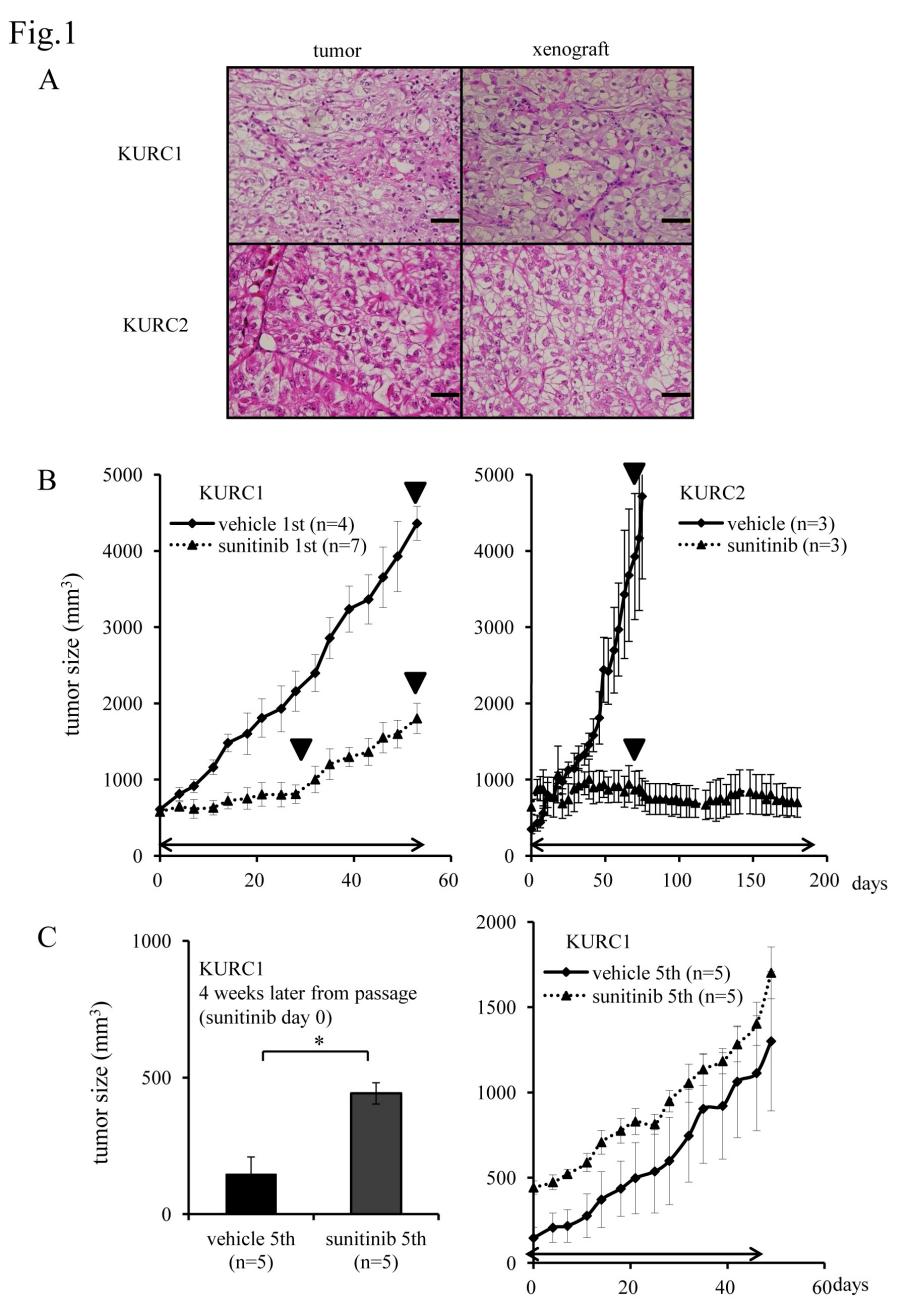
KURC1 tumors develop resistance to sunitinib but KURC2 remained sensitive. (A) Hematoxylin and eosin (H&E) staining of an original RCC surgical specimen and xenograft KURC1 and KURC2 tumor tissue. Scale bar, 50 μm. (B) The sequential changes of KURC1 and KURC2 xenograft tumors treated with sunitinib or vehicle only and (C) Left: KURC1 tumor volume at 4 weeks later from passage (sunitinib treatment day 0). Right: The sequential changes of KURC1 sunitinib 5^th^ and vehicle 5^th^. KURC1 repeatedly treated with sunitinib or vehicle 5^th^. Each time point represents the mean ± SE of tumor volume in each group. Day 0 is the first day of sunitinib administration at 4 weeks after transplantation. The difference in tumor size between sunitinib 5^th^ group and vehicle 5^th^ group in KURC1 was not statistically significant using two-way repeated ANOVA. Arrowed bars indicate the periods of sunitinib administration. ▼ indicates the time point when tumors were resected.

Two cohorts of primary xenografts were established and stably engrafted following three or more passages *in vivo*. We named them as primary xenograft KURC1 and KURC2. Histopathological diagnosis of primary tumor was clear cell type RCC of Grade 2 > 3, pT2N0M0 for KURC1 and clear cell type RCC, G3, pT3aN0M1 for KURC2. Each xenograft mostly recaptured the histopathological features of original tumors in tumor grade and architecture (Fig 1A). We have found necrosis in the center of tumors and N-cadherin was strongly expressed in both xenograft tumors (S1 Fig).

Upon treatment with sunitinib, tumor progression was observed in KURC1 after 4 weeks of treatment. Conversely, KURC2 retained sensitivity for more than 6 months (Fig 1B). Importantly, when KURC1 tumors that acquired sunitinib resistance were transferred to other SCID mice and treated with sunitinib again, the resistant phenotype was successfully reproduced. After this procedure was repeated five times, latency period of KURC1 sunitinib group (named as sunitinib 5^th^) was shorter than vehicle group (vehicle 5^th^), and the xenograft of sunitinib 5^th^ acquired a complete resistance to sunitinib with maintaining clear-cell histological appearance and exhibited a similar tumor growth curve to the xenograft treated with vehicle 5^th^ (Fig 1C).

### Interleukin-13 receptor, alpha 2 (IL13RA2) as a candidate molecule responsible for the acquired resistance to sunitinib

To clarify the mechanism of sunitinib resistance, we compared the gene expression profile of representative xenografts with sensitive and resistant status (GSE66346). KURC1 and KURC2 sensitive tumors treated with sunitinib were harvested at day 30 and 60, respectively (Fig 1B). Sunitinib-treated resistant KURC1 tumors were taken at day 50. Additionally, corresponding tumors treated with vehicle were harvested as a control and the ratio of expression levels of each gene under sensitive and resistant status to that of control was calculated (Fig 1B).

Three genes, IL13RA2, MTMR7 and FAM5B, were significantly highly expressed in sunitinib-resistant KURC1 xenografts (day 50) compared with those treated with vehicle (ratio = 6.50, 3.37, 2.95, respectively). Conversely, the expression levels of these genes were not significantly changed in sunitinib-sensitive KURC1 (day 30) and sunitinib-sensitive KURC2 compared with those in vehicle-treated KURC1 and KURC2 (Table 1).

**Table 1:** mRNA changes after sunitinib treatment in KURC1 and KURC2 xenograft tumors.

refseq	genedescription	genesymbol	KURC1R/C	KURC1S/C	KURC2S/C
NM_000640	interleukin 13 receptor, alpha 2	IL13RA2	6.50	0.99	1.45
NM_004686	myotubularin related protein 7	MTMR7	3.37	1.21	1.13
NM_021165	family with sequence similarity 5, member B	FAM5B	2.95	1.10	0.88

Microarray analysis was performed using Affymetrix GeneChip Human Gene 1.0 ST Arrays. These 3 genes were up-regulated more than 2.5-fold expression in sunitinib-resistant KURC1 tumor xenograft compared with control.

**Fig 2:**
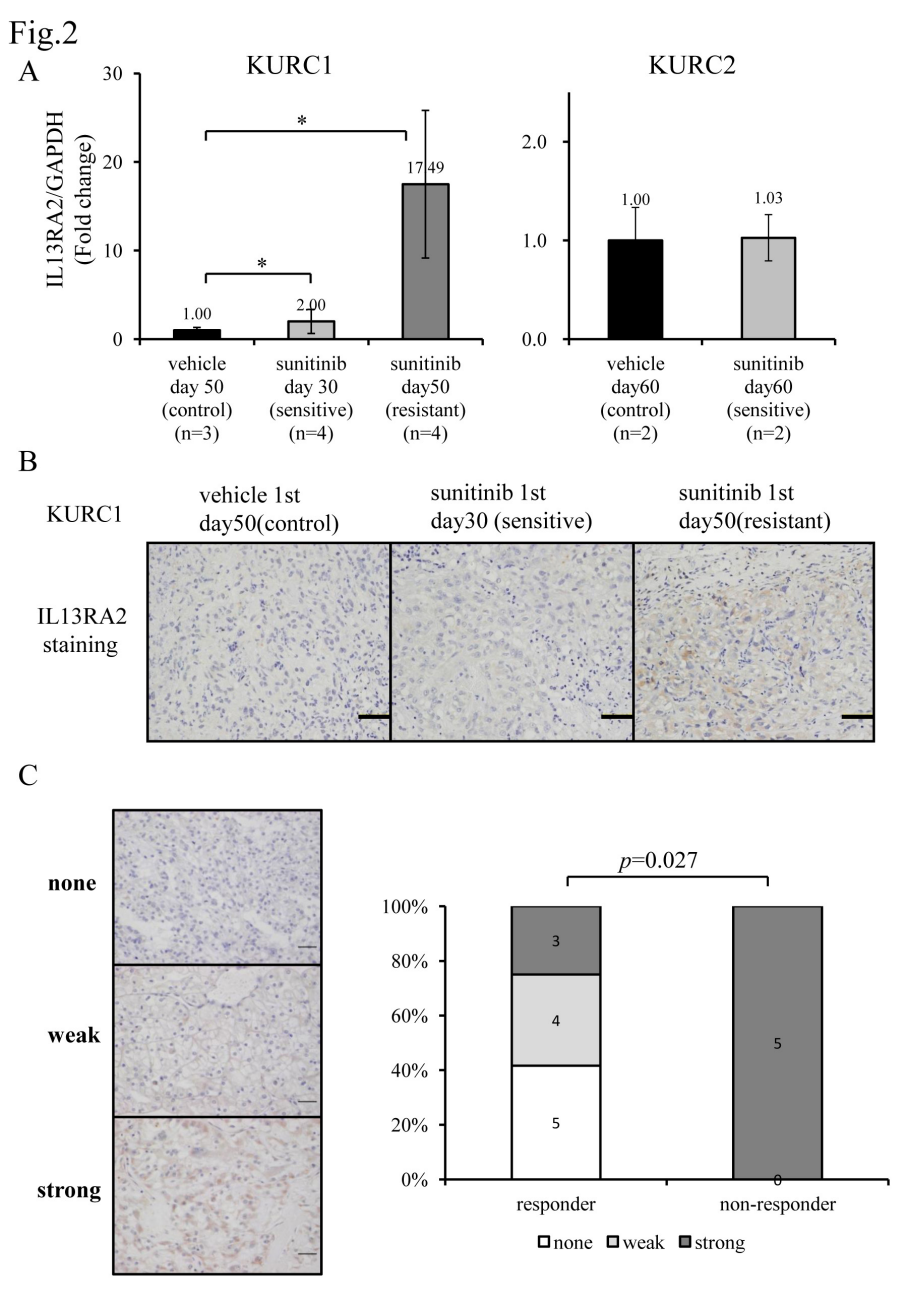
Evaluation of IL13RA2 mRNA and protein expression. (A) Evaluation of IL13RA2 mRNA expression in KURC1 and KURC2 xenograft tumors treated with sunitinib or vehicle by qPCR. All samples were prepared in triplicate and data are presented as the mean ± SE from indicated number of samples. Columns, mean; bar, SE. The difference in the mRNA expression levels between the sunitinib-treated group and control or sensitive group in KURC1 was statistically significant (*P < 0.01; Students’ *t*-test). There was no significant difference in KURC2 groups. (B) Immunohistochemical staining of IL13RA2 in KURC1 xenograft tumors. Scale bar, 50 μm. (C) IL13RA2 expression in human ccRCC tumors with the response to sunitinib treatment evaluated by immunohistochemistry. ccRCC tumor samples were collected from patients prior to sunitinib treatment. Left: representative pictures of immunohistochemistry sections of tumors showing none, weak, or strong staining for IL13RA2. Right: ratio of IL13RA2 expression pattern and correlation of the response to sunitinib treatment. Scale bar, 100 μm.

Among the three genes, qPCR analysis confirmed that IL13RA2 mRNA was significantly upregulated in sunitinib-resistant KURC1 compared with sunitinib-sensitive KURC1 or those treated with vehicles (Fig 2A and 2B). The other candidate genes, FAM5B and MTMR7, were also up-regulated in our experimental model of KURC1 and KURC2. But the expression was very low level, therefore we didn’t pick up these genes (S2 Fig).

Immunohistochemical analysis confirmed that the protein expression of IL13RA2 was upregulated in sunitinib-resistant KURC1 tumors compared with sunitinib-sensitive KURC1 tumors or those treated with vehicle (Fig 2B). KURC1 with complete resistance (mentioned above) showed extremely high IL13RA expression levels compared with those treated with vehicle alone (S3A and S3B Fig). The same analyses using KURC2 tumors showed no significant changes in IL13RA2 levels between sunitinib-treated and vehicle-treated tumors (Table 1, Fig 2A & S3C Fig).

Next, we examined IL13RA2 expression in human metastatic ccRCC tumors. Tumor samples were collected from primary lesion of metastatic ccRCC patients. These patients were subsequently treated with sunitinib and the response was evaluated by Response Evaluation Criteria in Solid Tumors guidelines. Five patients showed progressive disease within 3 month under sunitinib treatment (non-responder), whereas 12 patients showed partial response or stable disease over 3 month (responder). Although 3 patients were responder to sunitinib even high expression of IL13RA2 in their primary tumors, most patients with primary ccRCC tumors of higher IL13RA2 expression were non-responder group when they were treated with sunitinib for metastatic sites compared with those of lower IL13RA2 expression (Fig 2C). Collectively, these data suggested that IL13RA2 expression levels might be correlated to the sensitivity to sunitinib in ccRCC.

### Higher expression of IL13RA2 causes sunitinib resistance and tumor progression in ccRCC

**Fig 3:**
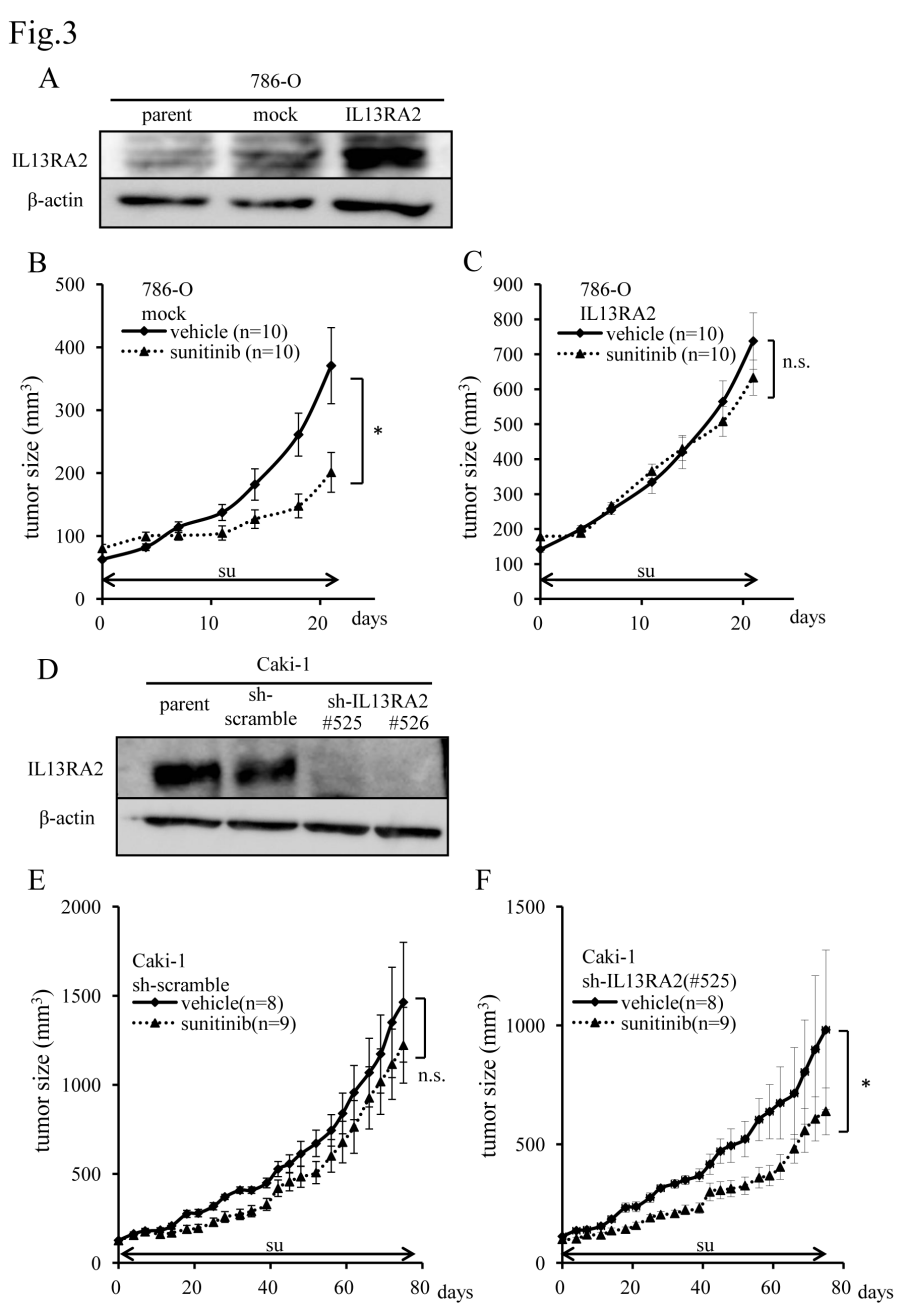
Overexpression of IL13RA2 leads to acquired resistance to sunitinib and shRNA-mediated IL13RA2 knockdown induces sensitivity to sunitinib. (A) Immunoblot analysis of 786-O subclones infected with retrovirus encoding mock or WT IL13RA2. Whole cell extracts were immunoblotted using the indicated antibodies. Sequential changes in subcutaneous xenograft tumors from 786-O subclones infected with (B) mock or (C) WT IL13RA2 treated with sunitinib and vehicle (control). Each time point represents the mean ± SE of tumor volume in each group. The difference in tumor size between the treatment group and control was statistically significant in 786-O-mock cells but not statistically significant in 786-O-IL13RA2 cells (*P < 0.05, n.s.: not significant; two-way repeated ANOVA). The horizontal arrow bars indicate the periods of sunitinib administration. (D) Immunoblot analysis of Caki-1 subclones infected with lentivirus encoding scrambled or IL13RA2 shRNA. Whole cell extracts were immunoblotted using the indicated antibodies. Sequential changes of subcutaneous xenograft tumors from a Caki-1 subclone infected with (E) scrambled or (F) IL13RA2 shRNA treated with sunitinib and vehicle (control). Each time point represents the mean ± SE of tumor volume in each group. Day 0 is the first day of sunitinib administration 4 weeks after transplantation. The difference in tumor size between the treatment group and control was not significant in Caki-1-sh-scrambled cells but statistically significant in Caki-1-sh-IL13RA2 cells (n.s.: not significant, *P < 0.05; two-way repeated ANOVA). The arrow bars indicate the period of sunitinib administration.

To clarify the effect of IL13RA2 on sunitinib sensitivity, sunitinib-sensitive 786-O cells [24] were infected with retrovirus encoding IL13RA2 or empty vector. The expression of exogenous IL13RA2 was confirmed by western blot (Fig 3A). Both cell lines were inoculated into nude mice to examine the sensitivity to sunitinib. IL13RA2 expression was still up-regulated in the xenograft tumors of IL13RA2-expressing 786-O (S4A Fig). Notably, the tumor growth of xenografts from IL13RA2-expressing 786-O cells was not suppressed by treatment with sunitinib (Fig 3C), while that of mock-transfected 786-O xenografts was significantly inhibited by sunitinib (Fig 3B). Moreover, tumor growth of IL13RA2-expressing 786-O xenografts was significantly more rapid than that of mock-transfected 786-O xenografts (S4B Fig).

To further confirm the effects of IL13RA2 on ccRCC, sunitinib-resistant Caki-1 cells [17] were infected with a lentivirus encoding scrambled or IL13RA2 shRNA. ShRNA-mediated knockdown of IL13RA2 successfully reduced the expression of IL13RA2 protein levels (Fig 3D). IL13RA2 expression was also maintained in the xenograft tumors (S5A Fig). Importantly, Caki-1 xenografts with IL13RA2 shRNA exhibited significantly higher sensitivity to sunitinib than those with scrambled shRNA (Fig 3E and 3F). Additionally, tumor growth *in vivo* was significantly suppressed by shRNA-mediated knockdown of IL13RA2 (S5B Fig).

Taken together, these results further supported the notion that IL13RA2 expression is associated with sensitivity to sunitinib and the biological aggressiveness of ccRCC.

### IL13RA2-mediated resistance to sunitinib is caused by the inhibition of sunitinib-induced apoptosis without increasing tumor microvasculature

**Fig 4:**
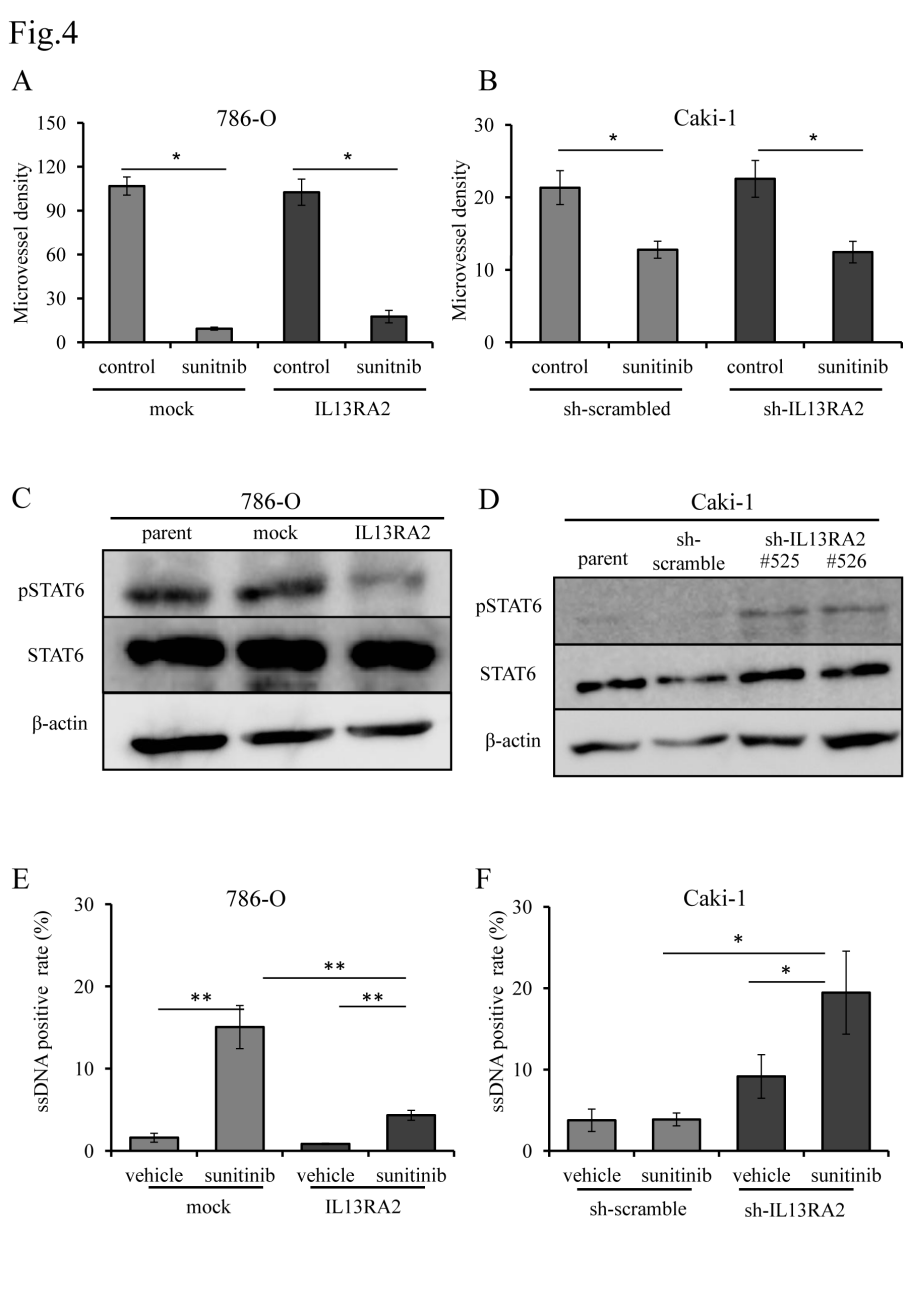
Evaluation of apoptosis and microvessel density after sunitinib treatment in xenograft model derived from cell lines. MVD was decreased by sunitinib treatment of each xenograft tumor derived from (A) 786-O or (B) Caki-1 subclones regardless of IL13RA2 expression level. MVD was determined from CD31 staining using Image J software. Statistical analysis was performed using the Students’ *t*-test (*P < 0.01). Immunoblot analysis of (C) 786-O subclones and (D) Caki-1 subclones. IL13RA2 expression was negatively correlated with the phosphorylation of STAT6. Whole cell extracts were immunoblotted using the indicated antibodies. ssDNA staining of xenograft tumors derived from (E) 786-O subclones and (B) Caki-1 subclones treated with sunitinib or vehicle only. Apoptosis was assessed by calculating the ssDNA positivity rate. Statistical analysis was performed using the Students’ *t*-test (*P < 0.05, **P < 0.01).

Next, we sought to explore the mechanisms of IL13RA2-mediated resistance to sunitinib. Xenograft tumors derived from 786-O and Caki-1 subclone were resected after 21days, 75days of sunitinib treatment, respectively. Since increased MVD was associated with sunitinib resistance [24, 25], we first measured MVD in a series of xenograft tumors from 786-O- or Caki-1-derived cells. As expected, MVD was significantly decreased in sunitinib-sensitive 786-O mock xenografts after the treatment (Fig 4A & S6A Fig). To our surprise, sunitinib significantly suppressed tumor microvasculature in specimens from resistant 786-O IL13RA2 cells (Fig 4A & S6A Fig). Similarly, CD31 immunostaining exhibited significantly decreased MVD in both sunitinib-treated sh-scrambled and shIL13RA2 Caki-1-derived xenografts irrespective of their sensitivity to the drug (Fig 4B & S6B Fig). These results indicated that IL13RA2 did not grossly affect the effect of sunitinib on tumor microvasculature and might play significant roles in the survival of tumor cells under the stressed circumstances of diminished MVD.

A previous study reported that higher expression of IL13RA2 was associated with tumor progression of certain types of cancer [26, 27]. Furthermore, IL13RA2 was shown to inhibit apoptotic cell death through the suppression of STAT6 phosphorylation by functioning as a decoy receptor for IL13 signaling. In fact, siRNA-mediated knockdown of IL13RA2 induced apoptosis in glioblastoma cells [28]. We next examined if IL13RA2 could suppress STAT6 phosphorylation in ccRCC cells. As shown in Fig 4C, the abundance of pSTAT6 was reduced in 786-O IL13RA2 cells compared with mock infected cells. This was also true in Caki-1 cells, in that shRNA-mediated knockdown of IL13RA2 resulted in the upregulation of phosphorylated STAT6 (Fig 4D).

Based on these results, we examined the effects of IL13RA2 on apoptotic cell death induced by sunitinib treatment *in vivo* by analyzing ssDNA positive rate in xenograft tumors with IL13RA2 overexpression. The positive ratio of ssDNA immunostaining was 15% when xenografts from mock-transfected 786-O cells were treated with sunitinib, and 1.5% in xenografts treated with vehicle. However, the positive ratio of ssDNA staining was only 4.3% when xenografts from IL13RA2-transfected 786-O cells were treated with sunitinib compared with 0.8% when treated with vehicle. Thus, xenografts from IL13RA2-expressing 786-O cells were more likely to escape from apoptosis by sunitinib treatment than mock-transfected 786-O cells (Fig 4E & S7A Fig).

As for xenograft tumors derived from Caki-1 cells, the number of apoptotic tumor cells indicated by ssDNA immunopositivity was not significantly increased by sunitinib treatment compared with vehicle in scramble shRNA-infected cells. However, in those derived from Caki-1 shIL13RA2 cells, apoptosis was significantly induced by sunitinib treatment compared with vehicle only (Fig 4F & S7B Fig).

**Fig 5:**
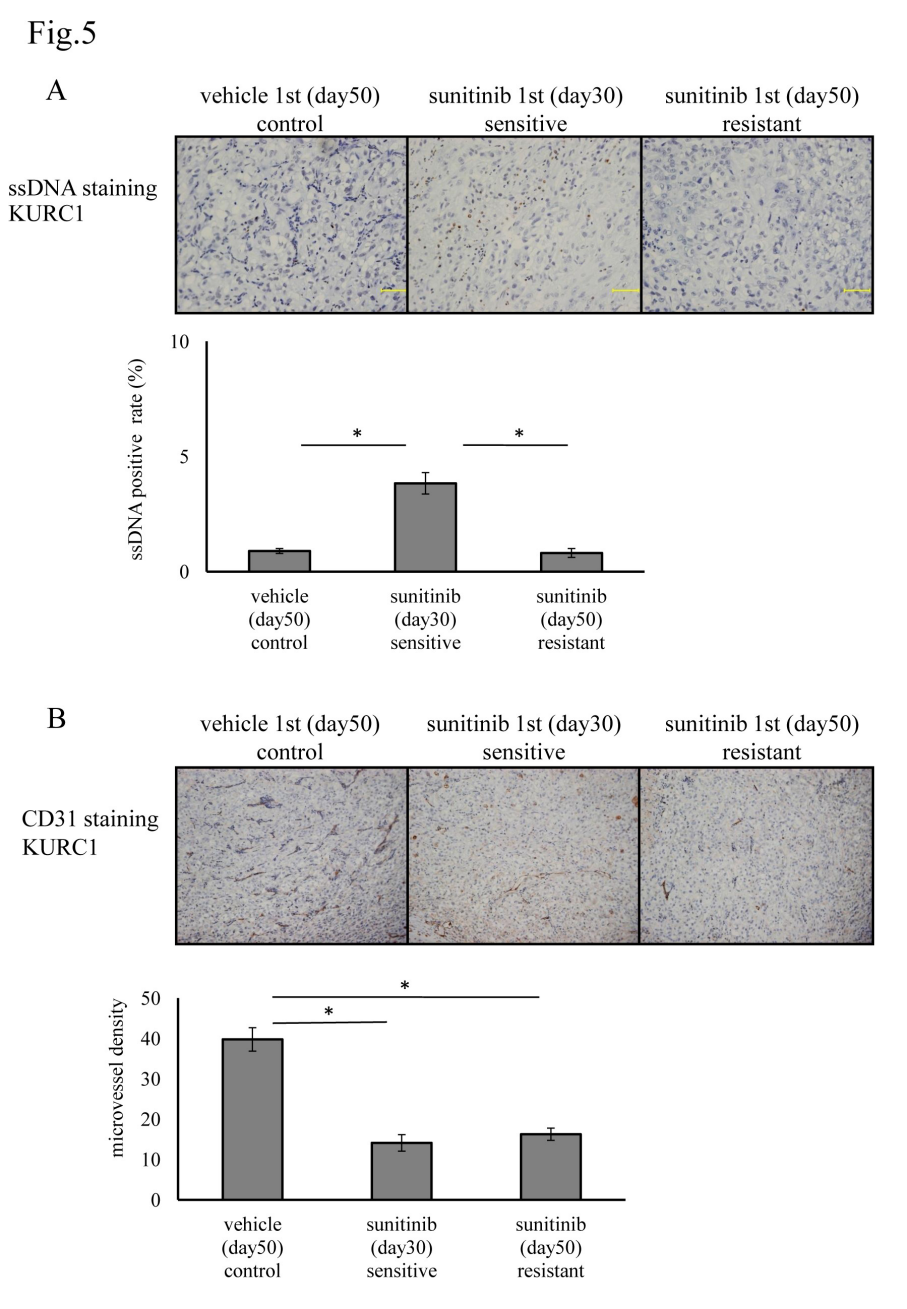
Evaluation of apoptosis and microvessel density after sunitinib treatment in our primary xenograft models. (A) ssDNA staining and (B) CD31 staining of KURC1 treated with sunitinib or vehicle with different sensitivity status. Scale bar, 50 μm. Apoptosis was assessed by calculating the ssDNA positivity rate, and MVD was determined from CD31 staining using Image J software. Statistical analysis was performed using the Students’ *t*-test (*P < 0.01).

Finally, we examined if the inhibition of apoptosis regulated the sensitivity and development of sunitinib resistance in our primary xenograft model KURC1. We first examined the number of ssDNA-positive cells in both xenografts. Sunitinib treatment significantly increased the number of ssDNA-positive apoptotic cells in sunitinib-sensitive in KURC1 tumors at day 30. In contrast, in KURC1 sunitinib-resistant tumors at day 50, the number of apoptotic cells decreased to a level almost comparable to that of vehicle-treated cells at day 50 (Fig 5A). We next measured MVD in each xenograft. MVD was reduced after the treatment of sunitinib at day 30 or 50 in KURC1 xenograft tumors, irrespective of sunitinib sensitivity (Fig 5B). In order to estimate the total number of tumor vessels, MDV multiply the corresponding tumor volume. According to calculations, the ratio of number of vessels in control tumor at day 50, sensitive tumor at day 30, and resistant tumor at day 50 were 17, 1, and 3, respectively. Indeed, the ratio of them in p5 control tumor at day 50 and p5 resistant tumor at day 50 were estimated 2 and 1. These observations implicated total number of tumor vessels was slightly increased when tumors acquired sunitinib resistance despite of the reduction of MVD, although angiogenesis was still inhibited by sunitinib even in complete resistance state in our primary xenograft model. Collectively, these data suggested that IL13RA2-mediated resistance to sunitinib in ccRCC might be primarily caused by the inhibition of apoptosis.

## Discussion

Most patients with metastatic or unresectable RCC are treated with angiogenesis inhibitors targeting VEGFR or inhibitors of mTORC1 [29]. In particular, sunitinib is considered to be the standard treatment option for RCC [30, 31]. Unfortunately, most metastatic RCCs eventually develop resistance to sunitinib [12]. Although some limited reports have addressed the molecular mechanisms of the development of resistance to sunitinib or other anti-angiogenic drugs, these mechanisms are not fully understood.

Several studies suggested that at least four molecular mechanisms mediate acquired resistance to VEGF-targeted anti-angiogenic treatment including sunitinib: 1) activation and/or upregulation of other pro-angiogenic signaling pathways, for example fibroblast growth factor 1 and 2 (FGF1/2), ephrin A1 and A2 (Efna1/2), and angiopoietin 1 (Ang1) [25]; 2) increased pericyte coverage of tumor blood vessels; 3) recruitment and survival of myeloid-derived suppressor cells with sustained immune suppression and angiogenesis[32, 33]; and 4) increased tumor cell invasiveness to escape from oxygen and nutrition deprivation[13, 25, 34]. Another study suggested that mechanisms of multidrug-resistance to chemotherapy in RCC might also be involved in decreased intake of tyrosine kinase inhibitors (TKIs), including those related with membrane structures, ATP-binding cassette (ABC) drug transporters with P-glycoprotein (P-gp, ABCB1), multidrug resistance associated protein (MRP) 1 (ABCC1), and ABCG2 (breast cancer resistance protein, MXR) [35]. Moreover, Gotink et al. introduced the idea of lysosomal sequestration as a specific cellular adaptation to toxic TKI concentrations in a TKI-resistant RCC *in vitro* model [36].

However, these proposed mechanisms do not fully address the process of acquired resistance to sunitinib in human RCC, because these mechanisms have not been validated in models that are well recapitulated for the clinical course of human RCCs. Furthermore, it is also difficult to compare human clinical tumor tissues before treatment with those after treatment.

Although human cancer cell lines are extremely useful for cancer research [37, 38], many have been passaged in culture for numerous years, resulting in alterations in their characteristics following adaptations to growth in culture and subsequent expansion [39, 40]. Thus, xenograft tumors derived from these cell lines often look quite different histologically from those routinely encountered in a clinical pathological laboratory. Therefore, in this study, we established two cohorts of primary xenograft models derived from patient surgical tissues that pathologically resembled the original tumors. Primary xenograft tumors derived from surgical specimens retain the histology as well as the gene expression levels, DNA copy number alterations and over 90% of the protein-coding gene mutations of the corresponding tumors [15]. These primary xenograft models are therefore considered to be reasonable models in which to recapitulate the clinical course of ccRCC treated with sunitinib.

In our primary xenograft models, KURC1 and KURC2 tumors displayed different characteristics regarding sunitinib sensitivity. KURC1 developed resistance to sunitinib after 4 weeks of treatment but KURC2 remained sensitive for more than 6 months. Although there have been some reports that primary xenograft tumors acquire resistance to sunitinib, previous studies showed that re-transplanted tumors in the next cohort of other mice recovered sensitivity to sunitinib [41]. Surprisingly, in our study, when resistant KURC1 tumors were resected, re-transplanted into other mice and repeatedly re-administered with sunitinib, KURC1 tumors finally demonstrated complete resistance to sunitinib after four passages. These different types of primary xenograft models (resistance-developing KURC1, sensitive KURC2, and completely resistant KURC1) were useful in our research in identifying novel mechanisms of development of sunitinib resistance.

To explore the underlying mechanisms of acquired resistance, we performed a microarray analysis comparing sunitinib-resistant and-sensitive KURC1 with controls, and sunitinib-sensitive KURC2 with controls. Additionally, we performed whole exome sequencing to compare completely sunitinib-resistant KURC1 with controls. Some gene expression changes were identified by microarray analysis, but acquired somatic mutations were not found in the exon regions within these genes of interest (unpublished data). Therefore, we compared gene expression changes in our data set with those in previous reports and evaluated whether these gene expression changes were related to sunitinib resistance.

The pro-angiogenic cytokine interleukin 8 (IL-8) was increased in the plasma of sunitinib-resistant xenograft mice, and neutralization of IL-8 blocked tumor angiogenesis and caused tumor re-sensitization to sunitinib [24, 42]. Reduction of IFNγ-related angiostatic chemokines and restoration of CXCL9 delayed acquired resistance to anti-VEGF treatment [43]. MMP1, SERPNTE1, ANGPTL4, NRP2, ARG2 and INSIG2 were elevated in sorafenib-resistant xenograft tumors derived from a 786-O cell line [44]. However, these gene levels were not upregulated in our primary xenograft model treated with sunitinib (S1 Table). These findings suggested that another mechanism is involved in acquired resistance to sunitinib in our primary xenograft KURC1 model.

IL13RA2 was identified as a candidate molecule associated with sunitinib resistance in our model KURC1, and IL13RA2 expression was significantly higher in human primary ccRCC tumor of patients with sunitinib-resistant metastatic sites. Even though 3 patients were responder to sunitinib whose primary RCC tumors were higher expression of IL13RA2, this molecule may be one candidate of sunitinib-resistance and other mechanism or molecules have been reported. Albeit, it would be better that we evaluated IL13RA2 expression level in the affected tumors after sunitinib treatment or after developing sunitinib-resistance, it is ethically difficult to obtain such samples. Therefore we evaluated that IL13RA2 expression in human primary tumors and their systemic response to sunitinib.

Besides, gene expression data derived from Oncomine reported by Vasselli indicated that ccRCC specimens of Furhman Grade 4 showed a significantly higher expression of IL13RA2 compared with those of Grade 3. Moreover, higher expression of this gene was also significantly associated with poor prognosis (dead at 1 year). In general, patients with high grade ccRCC tumors or patients of poor prognosis developed resistance to sunitinib earlier. These data might support our results. However other available dataset in ccRCC reported by Bittner showed IL13RA2 expression was not correlated with tumor grade, albeit it was slightly elevated in the cases of tumor Grade 4 (S8 Fig). Considering the number of Grade 4 cases was small in this data set, expression profile with more samples would be necessary to elucidate high IL13RA2 expression associate with worse clinical features.

IL13RA2 was originally cloned from the human renal cell carcinoma Caki-1 cell line [45]. This protein binds IL13 with high affinity but lacks a cytoplasmic domain and does not appear to function as a signal mediator, which is slightly dissimilar to interleukin 13 receptor, alpha 1 (IL13RA1) [46]. IL-13 and IL-4 modify the function of macrophages, and IL13RA2 mediates IL13/4 mediated tumor associated macrophage M1/M2-Th1/Th2 phenotype, especially, IL-13 signaling promote to change M1 to M2[47]. Tumor associated macrophage M2 promotes the epithelial-mesenchymal transition process in pancreatic cancer cells[48]. Our data was incompatible with these theories, therefore it may be important to evaluate the relationship of tumor microenvironment and IL13 signaling. Besides, IL13RA2 is associated with several types of cancer progression, including the EMT of prostate cancer [26, 27]. Furthermore, IL13 initiates an intracellular cascade that functions via 15-LOX-1 to activate PPAR-γ by phosphorylation of STAT6 and regulate cell proliferation and apoptosis. Silencing of IL13RA2 also promotes glioblastoma cell death by inducing apoptosis [28]. Together this indicates that upregulation of IL13RA2 inhibits apoptosis.

Generally, in 786-O tumor xenograft model, MVD in sensitive status was significantly decreased by sunitinib treatment, but after long term of sunitinib treatment MVD in resistant status was slightly increased in compared with sensitive status. This mechanism was suggested that other angiogenic factors (e.x. IL-8, FGF-1/2, Ang1 etc.) were elevated or induced by anti-angiogenic treatment [24].

In addition to these theories, our data suggested that IL13RA2 expression was correlated with tumor growth and sensitivity to sunitinib. Sunitinib failed to suppress tumor growth of 786-O xenograft tumors when IL13RA2 was overexpressed, and sunitinib inhibited tumor growth of Caki-1 xenograft tumors knocked down for IL13RA2 *in vivo*. Additionally, growth of xenograft tumors was promoted by upregulation of IL13RA2 and suppressed by silencing of IL13RA2. And moreover, MVD was also decreased by sunitinib treatment independent of IL13RA2 expression level. These data suggested that the mechanisms of acquired resistance related with IL13RA2 expression were not associated with re-angiogenesis in tumors despite the observations in previous reports [25, 49].

It was reported that tumor apoptosis was induced by upregulation of IL13RA2 in glioblastoma cells [28]. Another report showed that sunitinib induced apoptosis by inhibition of STAT3 [50]. In our model, overexpression of IL13RA2 inhibited tumor apoptosis induced by sunitinib and silencing of IL13RA2 promoted tumor apoptosis induced by sunitinib. These data suggested that IL13RA2 expression was related to acquired resistance to sunitinib through inhibiting tumor apoptosis, not via tumor re-angiogenesis. These findings were also seen in our primary xenograft model.

Recent studies showed that targeting IL13RA2 may be a new therapy for glioblastoma and head and neck squamous cell carcinoma [51, 52]. We conclude our observation suggests that IL13RA2 could mediate resistance to sunitinib in certain population of ccRCC by avoiding sunitinib-induced apoptosis. This is an unknown mechanism for acquiring resistance to sunitinib in addition to previous reports and suggests that IL13RA2 could be a potential target to help overcome sunitinib resistance.

## Supporting Information

S1 Fig N-cadherin staining of KURC1 and KURC2 xenograft tumors. N-cadherin staining of xenograft tumors derived from KURC1 and KURC2. Scale bar, 50 μm.
(PDF)

S2 Fig Evaluation of FAM5B and MTMR7 mRNA expression. Evaluation of FAM5B and MTMR7 mRNA expression in KURC1 and KURC2 xenograft tumors treated with sunitinib or vehicle by qPCR. All samples were prepared in triplicate and data are presented as the mean ± SE from indicated number of samples. Columns, mean; bar, SE. The difference in the mRNA expression levels between the sunitinib-treated group and control or sensitive group in KURC1 was statistically significant (*P < 0.01; Students’ *t*-test). There was no significant difference in KURC2 groups.
(PDF)

S3 Fig Evaluation of IL13RA2 mRNA and protein expression. (A) Evaluation of IL13RA2 mRNA expression in KURC1 xenograft tumors repeatedly treated with sunitinib or vehicle 5^th^ by qPCR. Columns, mean; bar, SE. The difference in the mRNA expression levels between the sunitinib-treated group and vehicle group in KURC1 was statistically significant (*P < 0.01; Students’ *t*-test). (B) Immunohistochemical staining of IL13RA2 in KURC1 xenograft tumors repeatedly treated with sunitinib 5^th^ or vehicle 5^th^. Scale bar, 50 μm. (C) Immunohistochemical staining of IL13RA2 in KURC2 xenograft tumors. Scale bar, 50 μm.
(PDF)

S4 Fig Xenograft tumor growth of 786-O subclone. (A) IL13RA2 staining of xenograft tumors derived from 786-O subclone overexpressing IL13RA2 or mock control. The difference of IL13RA2 expression level was maintained *in vivo*. (B) Xenograft tumor growth of 786-O subclone overexpressing IL13RA2 was increased compared with mock control. Day 0 is the day of transplantation. The difference was statistically significant (*P < 0.01; two-way repeated ANOVA).
(PDF)

S5 Fig Xenograft tumor growth of Caki-1 subclone. (A) IL13RA2 staining of xenograft tumors derived from Caki-1 subclone suppressed by shRNA-mediated knockdown of IL13RA2 and sh-scrambled subclones. The difference of IL13RA2 expression level was maintained *in vivo*. (B) Xenograft tumor growth of Caki-1 subclone was suppressed by shRNA-mediated knockdown of IL13RA2 (#525 and #526) compared with sh-scrambled subclones. Day 0 is the day of transplantation. The difference was statistically significant (*P < 0.01; two-way repeated ANOVA).
(PDF)

S6 Fig CD31 staining of cell line derived xenograft tumors. CD31 staining of xenograft tumors derived from (A) 786-O subclones and (B) Caki-1 subclones treated with sunitinib or control. Scale bar, 50 μm.
(PDF)

S7 Fig ssDNA staining of cell line derived xenograft tumors. ssDNA staining of xenograft tumors derived from (A) 786-O subclones and (B) Caki-1 subclones treated with sunitinib or control. Scale bar, 50 μm.
(PDF)

S8 Fig Gene expression data obtained from Oncomine. Data originated from Vasselli et al. (21). Left: IL13RA2 mRNA expression of Grade 3 ccRCC versus Grade 4. Right: IL13RA2 mRNA expression of ccRCC patients alive at 1 year versus those dead at 1 year. Lower: Data originated from Bittner IL13RA2 mRNA expression of ccRCC grouped by tumor grade.
(PDF)

S1 Table mRNA changes after sunitinib treatment in KURC1 and KURC2 xenograft tumors. Microarray analysis were performed using Affymetrix GeneChip Human Gene 1.0 ST Arrays. The 8 genes were previous report related with resistance to sunitinib or sorafenib in RCC.
(XLSX)
