# Supplementary figures and images for: Role of IL13RA2 in Sunitinib Resistance in Clear Cell Renal Cell Carcinoma
Source: PLoS One. 2015 Jun 26;10(6):e0130980. doi: 10.1371/journal.pone.0130980 (PMC4482605; doi:10.1371/journal.pone.0130980)

S1 Fig.

N-cadherin  
staining

KURC1

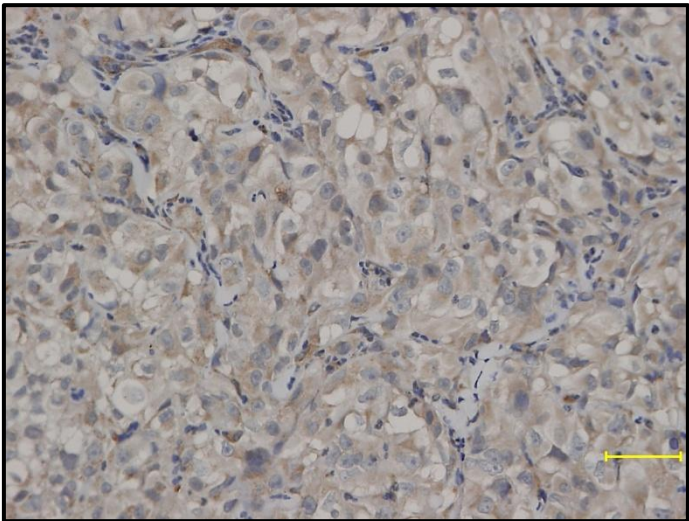

KURC2

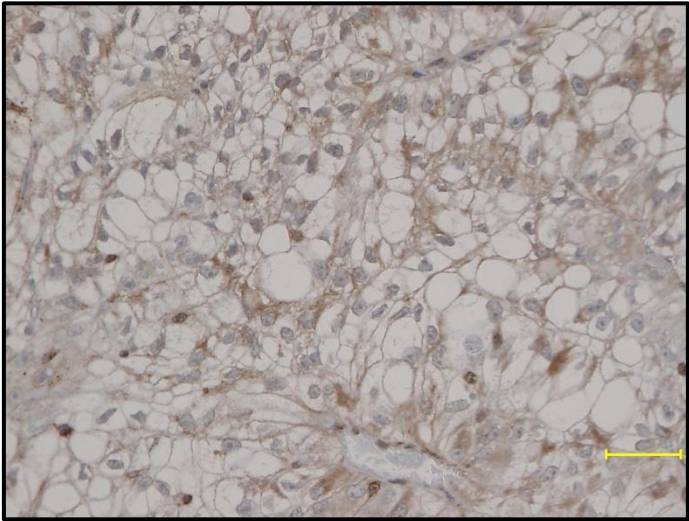

Supplement: S1 Fig — N-cadherin staining of xenograft tumors derived from KURC1 and KURC2. Scale bar, 50 μm. (PDF) [file pone.0130980.s001.pdf]

S2 Fig.

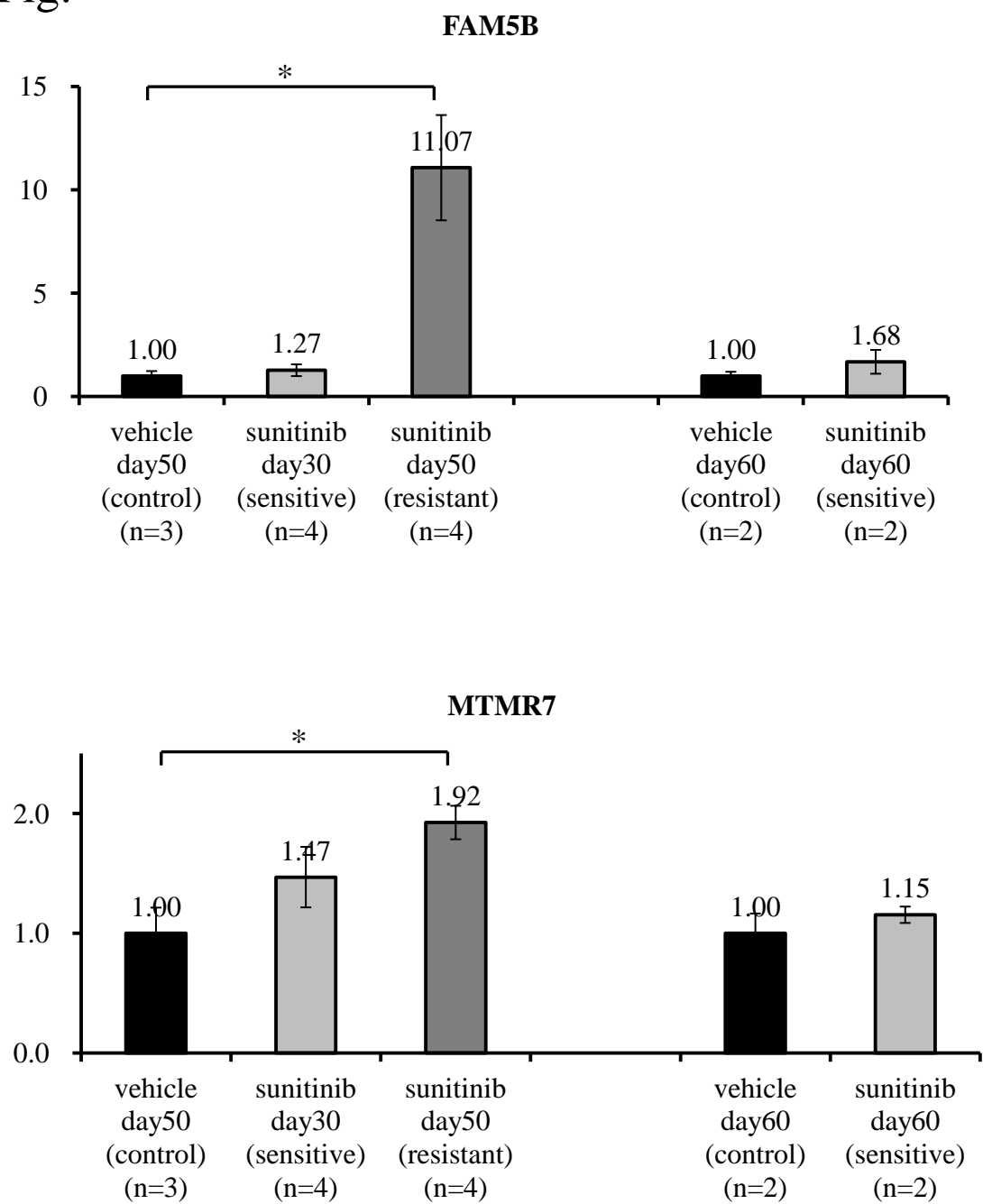

Supplement: S2 Fig — Evaluation of FAM5B and MTMR7 mRNA expression in KURC1 and KURC2 xenograft tumors treated with sunitinib or vehicle by qPCR. All samples were prepared in triplicate and data are presented as the mean ± SE from indicated number of samples. Columns, mean; bar, SE. The difference in the mRNA expression levels between the sunitinib-treated group and control or sensitive group in KURC1 was statistically significant (*P < 0.01; Students’ t-test). There was no significant difference in KURC2 groups. (PDF) [file pone.0130980.s002.pdf]

S3 Fig.

A

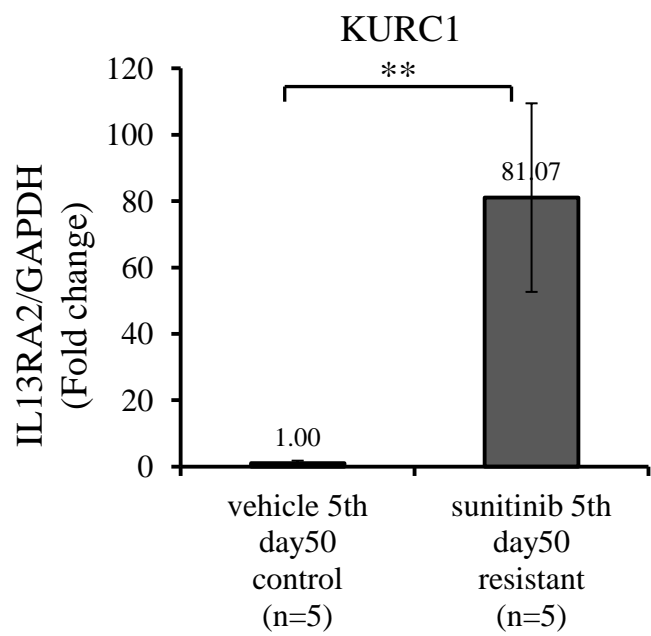

B

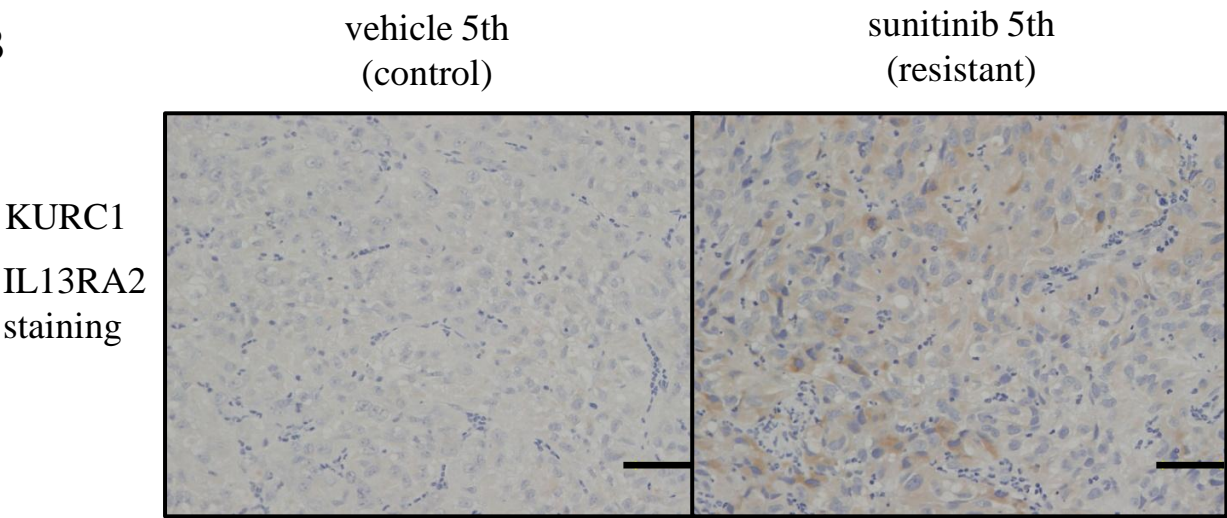

C

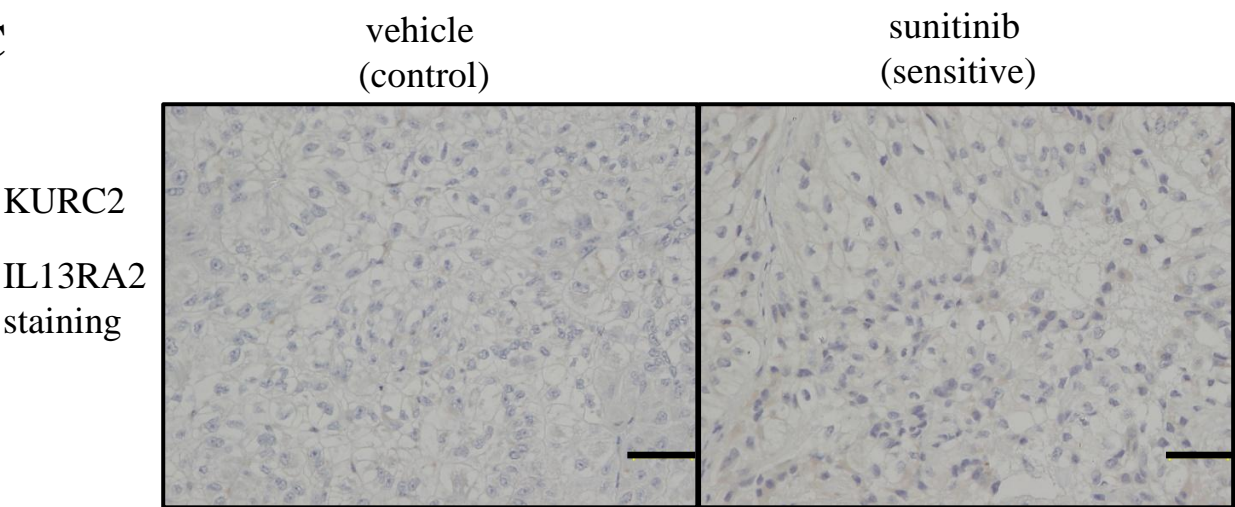

Supplement: S3 Fig — (A) Evaluation of IL13RA2 mRNA expression in KURC1 xenograft tumors repeatedly treated with sunitinib or vehicle 5th by qPCR. Columns, mean; bar, SE. The difference in the mRNA expression levels between the sunitinib-treated group and vehicle group in KURC1 was statistically significant (*P < 0.01; Students’ t-test). (B) Immunohistochemical staining of IL13RA2 in KURC1 xenograft tumors repeatedly treated with sunitinib 5th or vehicle 5th. Scale bar, 50 μm. (C) Immunohistochemical staining of IL13RA2 in KURC2 xenograft tumors. Scale bar, 50 μm. (PDF) [file pone.0130980.s003.pdf]

S4 Fig.

A

IL13RA2  
staining

786-O

Mock

IL13RA2

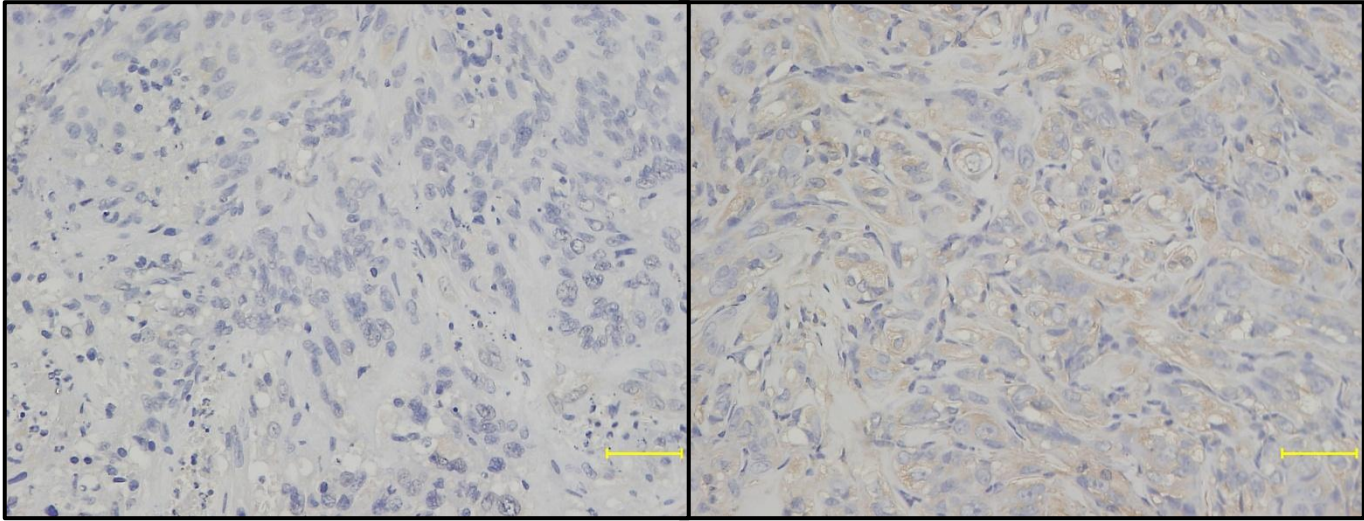

B

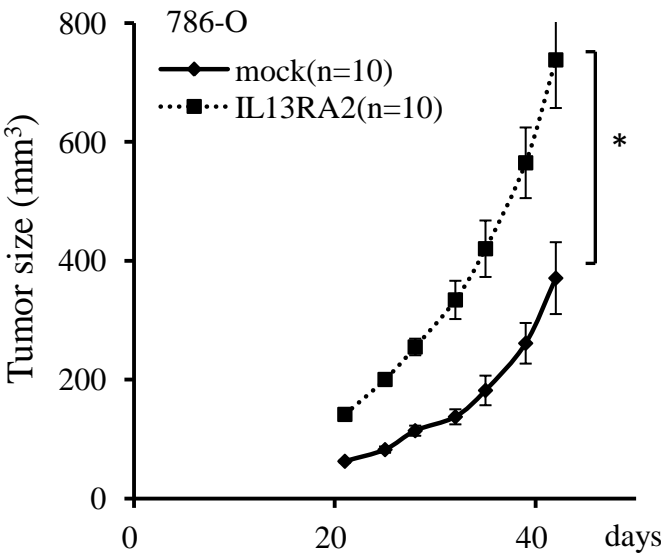

Supplement: S4 Fig — (A) IL13RA2 staining of xenograft tumors derived from 786-O subclone overexpressing IL13RA2 or mock control. The difference of IL13RA2 expression level was maintained in vivo. (B) Xenograft tumor growth of 786-O subclone overexpressing IL13RA2 was increased compared with mock control. Day 0 is the day of transplantation. The difference was statistically significant (*P < 0.01; two-way repeated ANOVA). (PDF) [file pone.0130980.s004.pdf]

S5 Fig.

A

IL13RA2  
staining

Caki-1

sh-scrambled

sh-IL13RA2

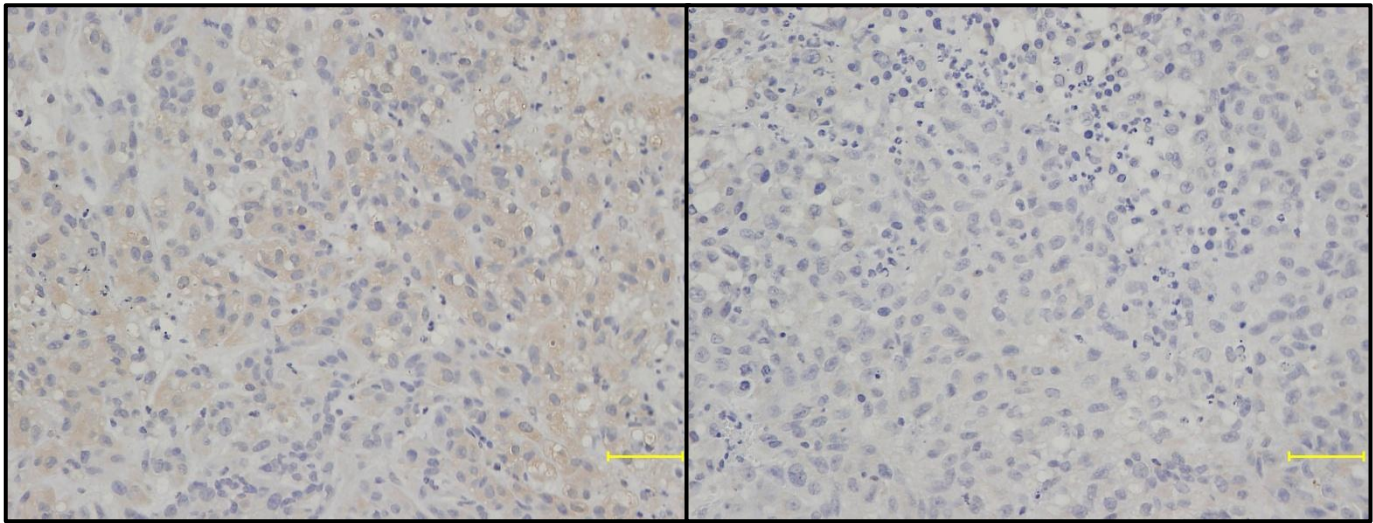

B

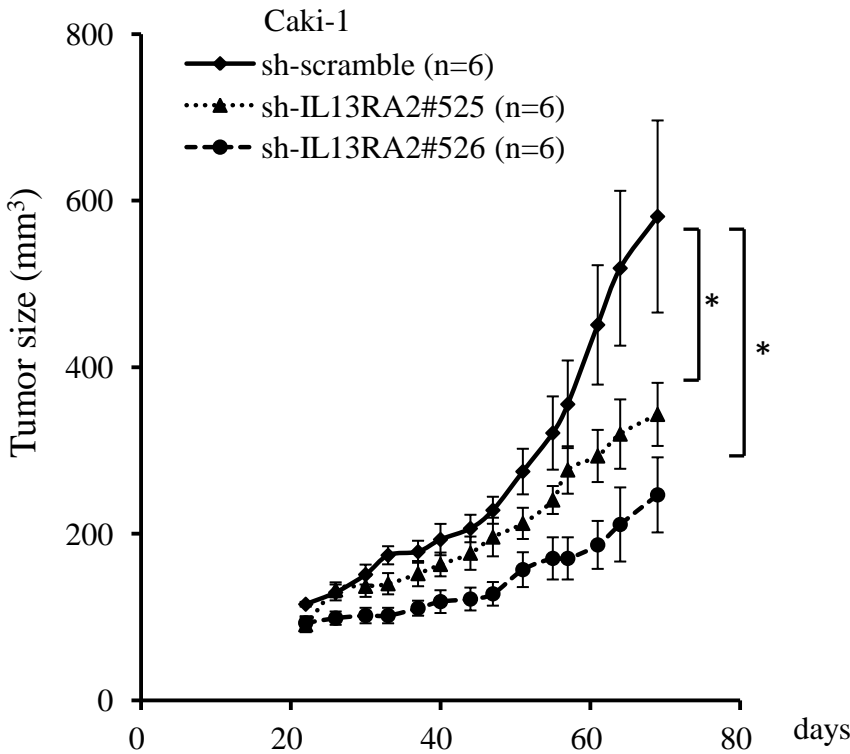

Supplement: S5 Fig — (A) IL13RA2 staining of xenograft tumors derived from Caki-1 subclone suppressed by shRNA-mediated knockdown of IL13RA2 and sh-scrambled subclones. The difference of IL13RA2 expression level was maintained in vivo. (B) Xenograft tumor growth of Caki-1 subclone was suppressed by shRNA-mediated knockdown of IL13RA2 (#525 and #526) compared with sh-scrambled subclones. Day 0 is the day of transplantation. The difference was statistically significant (*P < 0.01; two-way repeated ANOVA). (PDF) [file pone.0130980.s005.pdf]

S6 Fig.

A

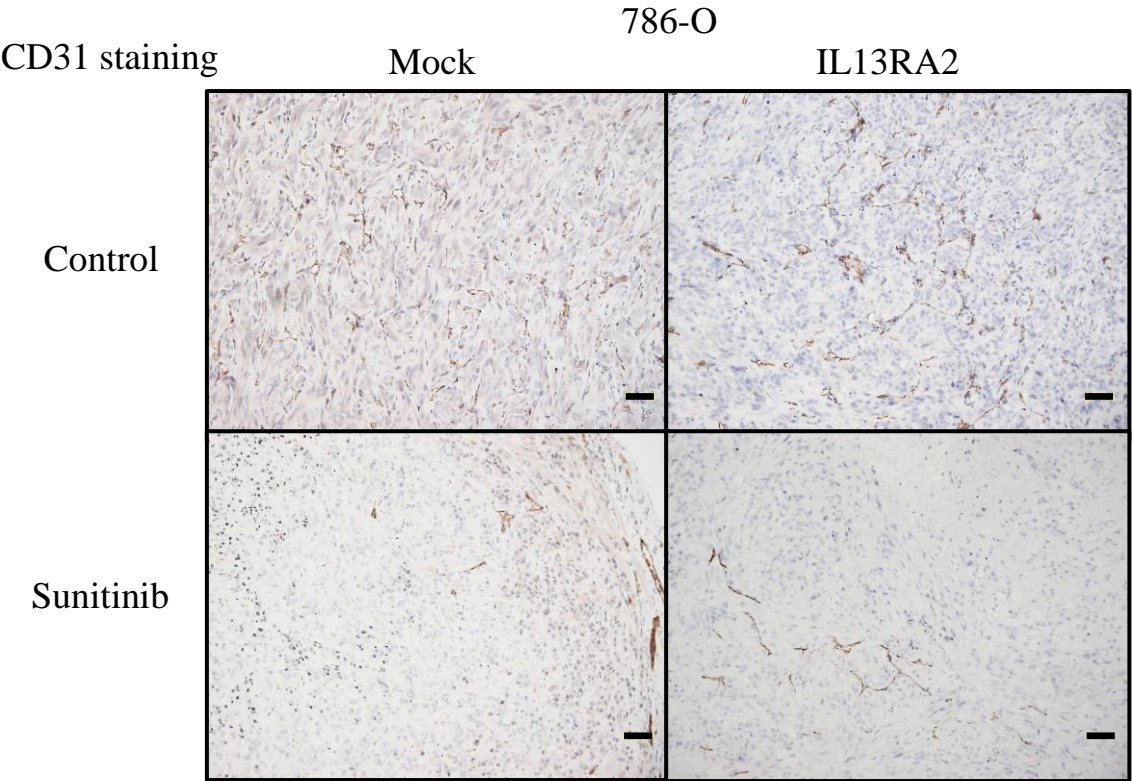

B

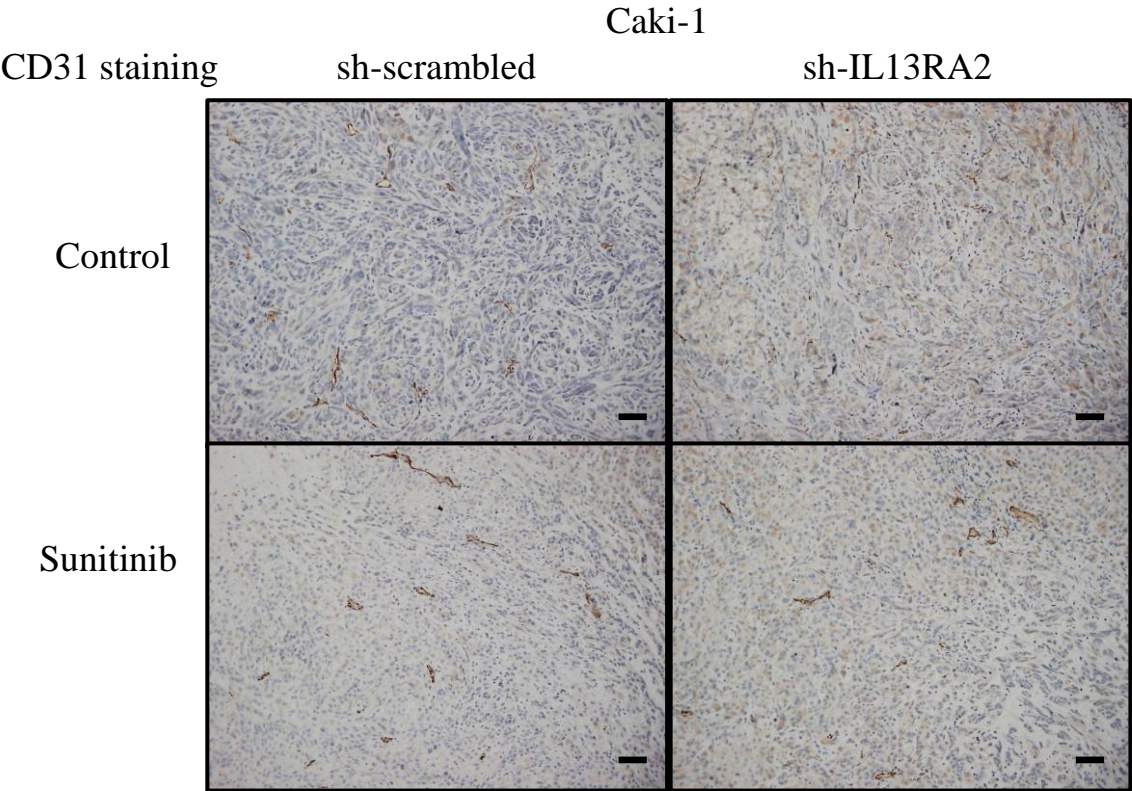

Supplement: S6 Fig — CD31 staining of xenograft tumors derived from (A) 786-O subclones and (B) Caki-1 subclones treated with sunitinib or control. Scale bar, 50 μm. (PDF) [file pone.0130980.s006.pdf]

S7 Fig.

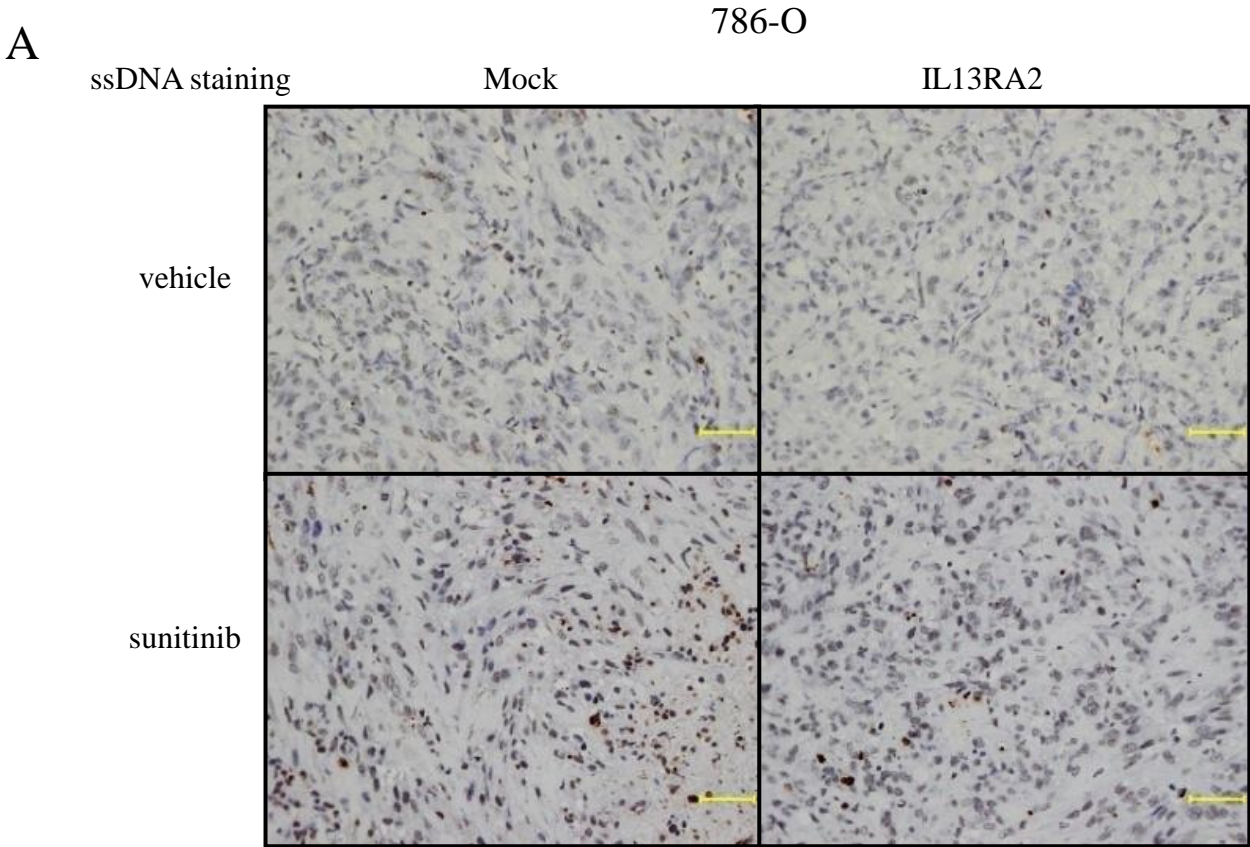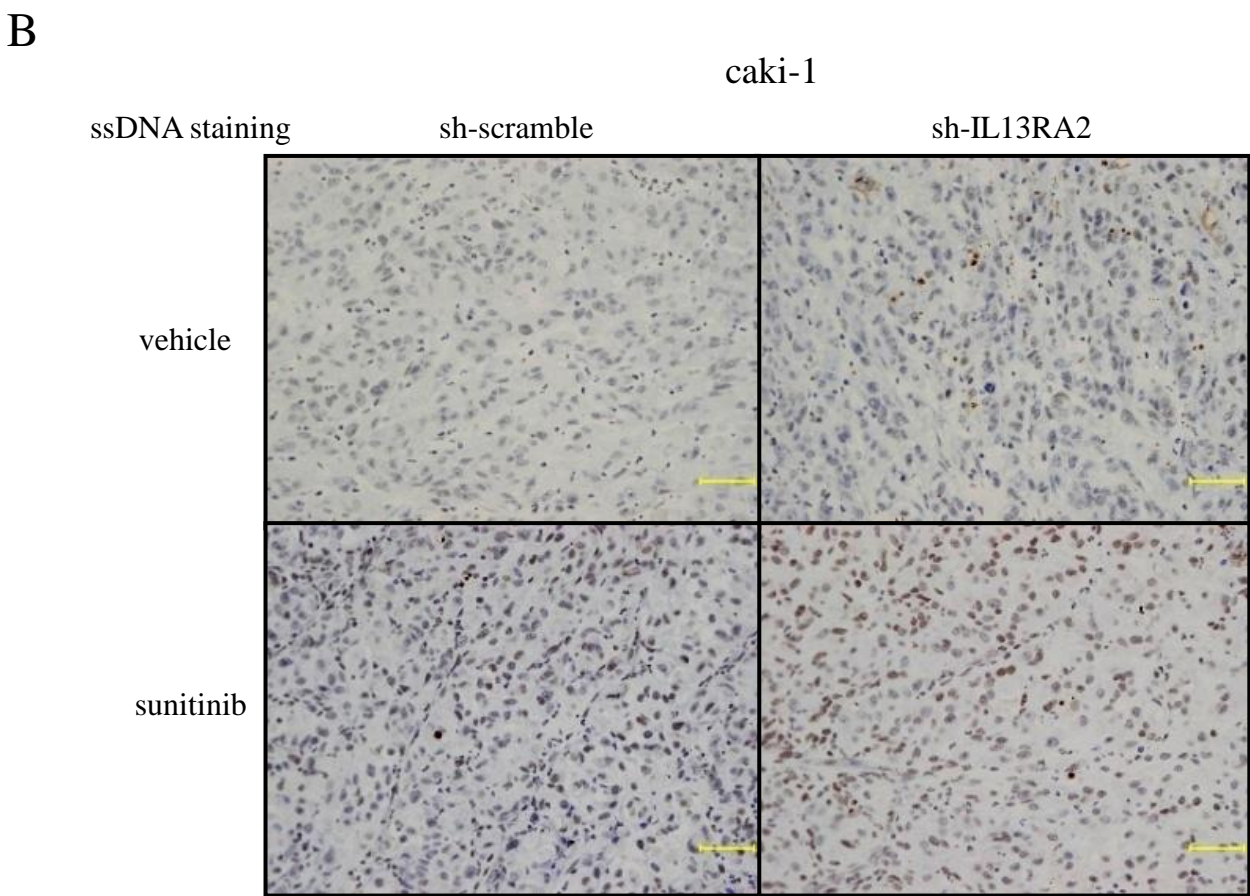

Supplement: S7 Fig — ssDNA staining of xenograft tumors derived from (A) 786-O subclones and (B) Caki-1 subclones treated with sunitinib or control. Scale bar, 50 μm. (PDF) [file pone.0130980.s007.pdf]
