# Supplementary material for: Role of IL13RA2 in Sunitinib Resistance in Clear Cell Renal Cell Carcinoma
Source: PLoS One. 2015 Jun 26;10(6):e0130980. doi: 10.1371/journal.pone.0130980 (PMC4482605; doi:10.1371/journal.pone.0130980)
Supplement: S8 Fig — Data originated from Vasselli et al. (21). Left: IL13RA2 mRNA expression of Grade 3 ccRCC versus Grade 4. Right: IL13RA2 mRNA expression of ccRCC patients alive at 1 year versus those dead at 1 year. Lower: Data originated from Bittner IL13RA2 mRNA expression of ccRCC grouped by tumor grade. (PDF) [file pone.0130980.s008.pdf]

S8 Fig.

IL13RA2 mRNA expression in clear cell renal cell carcinoma reported by Vasselli

Grade 3 versus Grade 4

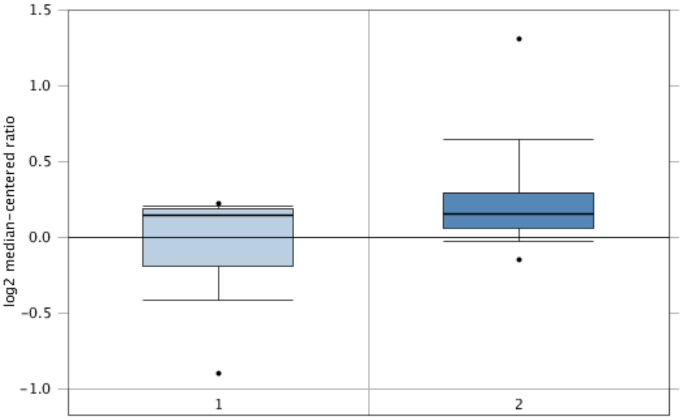

Grade 3 (n=12) Grade 4 (n=29)

***P*=0.011**

Alive at 1 year versus dead at 1 year

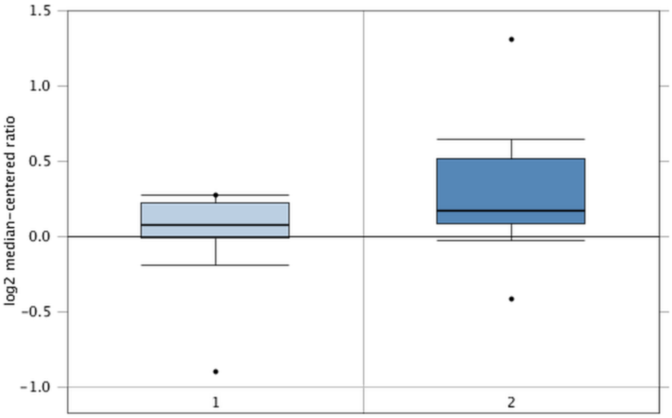

Alive at 1 year (n=20) Dead at 1 year (n=21)

***P*=0.015**

IL13RA2 mRNA expression in clear cell renal cell carcinoma reported by Bittner

Grade 1 ~ 4

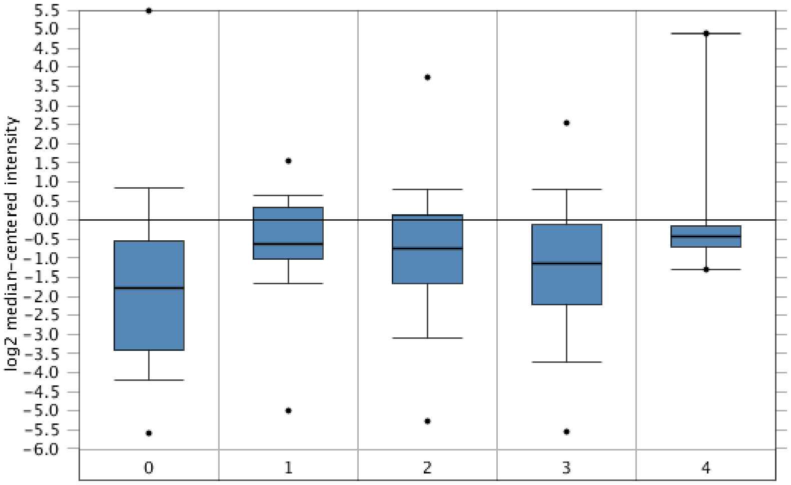

No value (n=86) Grade 1 (n=20) Grade 2 (n=104) Grade 3 (n=38) Grade 4 (n=8)
